# Ablation as targeted perturbation to rewire communication network of persistent atrial fibrillation

**DOI:** 10.1371/journal.pone.0179459

**Published:** 2017-07-05

**Authors:** Susumu Tao, Samuel F. Way, Joshua Garland, Jonathan Chrispin, Luisa A. Ciuffo, Muhammad A. Balouch, Saman Nazarian, David D. Spragg, Joseph E. Marine, Ronald D. Berger, Hugh Calkins, Hiroshi Ashikaga

**Affiliations:** 1 Cardiac Arrhythmia Service, Johns Hopkins University School of Medicine, Baltimore, Maryland, United States of America; 2 Department of Computer Science, University of Colorado, Boulder, Colorado, United States of America; 3 Santa Fe Institute, Santa Fe, New Mexico, United States of America; 4 Section for Cardiac Electrophysiology, University of Pennsylvania Perelman School of Medicine, Philadelphia, Pennsylvania, United States of America; 5 Department of Biomedical Engineering, Johns Hopkins University School of Medicine, Baltimore, Maryland, United States of America; Universiteit Gent, BELGIUM

## Abstract

Persistent atrial fibrillation (AF) can be viewed as disintegrated patterns of information transmission by action potential across the communication network consisting of nodes linked by functional connectivity. To test the hypothesis that ablation of persistent AF is associated with improvement in both local and global connectivity within the communication networks, we analyzed multi-electrode basket catheter electrograms of 22 consecutive patients (63.5 ± 9.7 years, 78% male) during persistent AF before and after the focal impulse and rotor modulation-guided ablation. Eight patients (36%) developed recurrence within 6 months after ablation. We defined communication networks of AF by nodes (cardiac tissue adjacent to each electrode) and edges (mutual information between pairs of nodes). To evaluate patient-specific parameters of communication, thresholds of mutual information were applied to preserve 10% to 30% of the strongest edges. There was no significant difference in network parameters between both atria at baseline. Ablation effectively rewired the communication network of persistent AF to improve the overall connectivity. In addition, successful ablation improved local connectivity by increasing the average clustering coefficient, and also improved global connectivity by decreasing the characteristic path length. As a result, successful ablation improved the efficiency and robustness of the communication network by increasing the small-world index. These changes were not observed in patients with AF recurrence. Furthermore, a significant increase in the small-world index after ablation was associated with synchronization of the rhythm by acute AF termination. In conclusion, successful ablation rewires communication networks during persistent AF, making it more robust, efficient, and easier to synchronize. Quantitative analysis of communication networks provides not only a mechanistic insight that AF may be sustained by spatially localized sources and global connectivity, but also patient-specific metrics that could serve as a valid endpoint for therapeutic interventions.

## Introduction

Atrial fibrillation (AF) currently impacts the lives of 3–6 million Americans, and the prevalence is expected to increase to 12 million by 2030 [1]. Invasive catheter ablation with pulmonary vein isolation (PVI) is superior to antiarrhythmic drugs [2] in maintaining sinus rhythm, and has become the standard approach to treating AF [3]; however, for persistent AF (continuous AF that sustains longer than 7 days) [4], PVI remains far from curative, with recurrence rates up to 40% [5]. Observational studies show that PVI [3], posterior wall isolation [6,7], a “stepwise” approach [8–10], and the Cox-Maze procedure [11] are at least partially effective in suppressing persistent AF. In essence, all of these strategies reduce the mass of contiguous atrial tissue below a “critical mass” needed to sustain reentry [12,13] by segmenting the atria with linear ablation lesions. A prospective randomized study, however, showed that linear lesions in addition to PVI do not improve the recurrence rates compared with PVI alone [5]. This important finding suggests that the geometry of atrial segmentation may need to be individually optimized based on patient-specific metrics, because each patient may have a different critical mass (“effective size”) [14] defined by tissue-specific conduction velocity and refractoriness.

The heart is a communication system [15] where action potential propagation allows information to transmit across the functional network, consisting of nodes (groups of cardiomyocytes) linked by functional connectivity (shared information). Persistent AF can be viewed as disintegrated patterns of information transmission within the communication network, characterized by functional reentry circuits resulting from conduction block [16,17] and heterogeneous refractoriness associated with atrial fibrosis [18]. Sinus rhythm requires synchronous excitation of the communication network, which is facilitated by a high degree of both local and global connectivity. High local connectivity requires that adjacent nodes communicate directly with each other (high clustering), while high global connectivity requires that remote nodes communicate with each other through a small number of nodes (short path length) [19–24]. Both high clustering and short path length are important features of small-world networks [25] by analogy with the small-world phenomenon of strangers linked by a mutual acquaintance [26]. The small-world network topology is found in a broad range of systems including the U.S. electrical power grid, the collaboration network among movie actors, and the brain network of *C*. *elegans* [25]. Importantly, structural and functional networks of the human brain exhibit small-world attributes, and disruption of small-world topology is associated with disturbances in cognition and behavior, such as schizophrenia [27–31]. Therefore, quantitative assessment of the communication networks of AF may provide personalized metrics to measure the ability to suppress AF and to promote synchronous excitation that can be used to define the endpoint of ablation procedures.

In this study, we hypothesized that catheter ablation of persistent AF is associated with improvement in both local and global connectivity within the communication networks that account for the clinical benefits. To test this hypothesis, we performed multi-electrode recordings before and after the focal impulse and rotor modulation (FIRM)-guided ablation within each atrium using a 64-electrode basket catheter that maps a wide area of the atrium. We defined communication networks of persistent AF by nodes and edges; nodes correspond to the cardiac tissue adjacent to each electrode; the edges correspond to the amount of information shared between pairs of nodes [15]. We demonstrate that successful ablation effectively rewires the communication networks of persistent AF to increase the local clustering, decrease the global path length, and improve the small-world topology, suggesting more robust and efficient information transmission.

## Materials and methods

Please refer to the *Detailed Methods* section (Appendix in S1 File, Supporting information).

### Patient population

We enrolled 37 consecutive patients in the study who underwent catheter ablation of persistent AF with multi-electrode mapping. The protocol was approved by the Johns Hopkins Medicine Institutional Review Board, and all participants provided written informed consent.

### Multi-electrode mapping and catheter ablation

**Fig 1:**
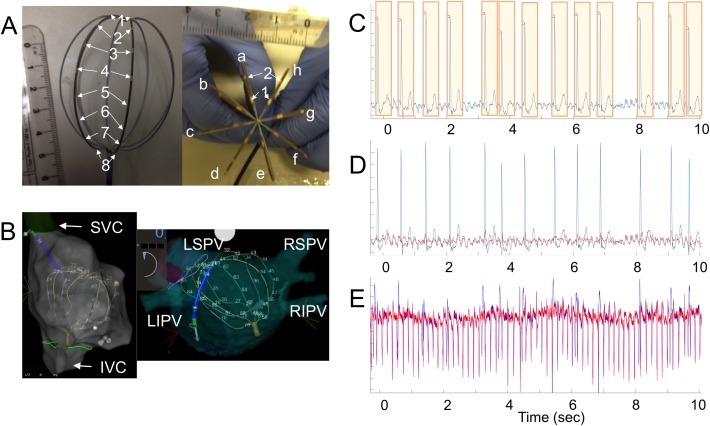
Multi-electrode mapping. ***A*.** *64-lead multi-electrode basket catheter*. The catheter has 8 electrodes in each spline, 9–11 mm apart, named 1 through 8 from the distal to the proximal apex (left panel). Each basket catheter has 8 splines, 45 degree apart, named *a* through *h* counterclockwise as views from the distal apex (right panel). ***B*.** *Basket catheter configuration in the atrium*. The basket catheter was inserted into the femoral vein and advanced to the right atrium (left panel, right anterior oblique view) through the inferior vena cava (IVC). Then the basket catheter was advanced trans-septally into the left atrium (right panel, posterior-anterior view). In both panels, the electroanatomical shell of each atrium demonstrates that the catheter covers a wide field of view: LSPV, left superior pulmonary vein; LIPV, left inferior pulmonary vein; RSPV, right superior pulmonary vein; LIPV, left inferior pulmonary vein. ***C*.** *Surface electrocardiogram (ECG) during atrial fibrillation (AF)*. The peak R wave (red circle) and the QRS-T complex (orange rectangle) were identified, and the mean QRS-T complex was computed from all the QRS complexes during the recording period. ***D*.** *Removal of ventricular signals from surface ECG*. The atrial ECG (red) was obtained by subtracting the mean QRS-T complex at each QRS-T complex of the surface ECG (blue) during the recording period. ***E*.** *Removal of far-field ventricular signals from basket catheter recordings*. Atrial electrogram (red) from the basket catheter recordings (blue) was obtained by the same method as above.

A 64-electrode basket catheter (50 or 60 mm; Abbott Electrophysiology, Menlo Park, CA; Fig 1A) was advanced to the right atrium (Fig 1B). Unipolar electrograms from the basket catheter were filtered at 0.05 to 500 Hz and recorded during AF for 60 seconds at a sampling frequency of 977 Hz (Cardiolab; GE Healthcare, Waukesha, WI). The unipolar electrograms were analyzed using the RhythmView workstation (Abbott Electrophysiology), and patients underwent radiofrequency (RF) catheter ablation based on the focal impulse and rotor modulation (FIRM)-guided approach [32,33]. Ablation was performed using a 3.5-mm tip, open-irrigated, force-sensing ablation catheter (ThermoCool SmartTouch; Biosense Webster, Inc., Diamond Bar, CA), targeting each area identified as a rotor center with power at the discretion of the operator. A repeat rotor map was obtained and any additional identified rotors were ablated [34]. The basket catheter was then advanced trans-septally to the left atrium, and multi-electrode mapping and ablation were repeated. After completion of the FIRM-guided ablation, pulmonary vein isolation was performed using wide-area circumferential ablation of the pulmonary vein antra until the entrance and exit block was demonstrated for each pulmonary vein.

### Clinical follow-up

Arrhythmic recurrence was defined as AF, atrial tachycardia, or atrial flutter of at least 30-second duration after a three-month blanking period [3]. At each in-office visit scheduled at 3 months after the ablation procedure, a physical examination and a 12-lead electrocardiogram (ECG) were performed. If symptoms suggestive of an arrhythmia occurred, patients were asked to undergo a 24-hour Holter monitor or a 30-day event monitor. Recurrence was defined at 6 months post-procedure.

### Mutual information

We performed data analysis using MATLAB R2016a (MathWorks, Inc., Natick, MA). After the far-field QRS-T complexes were removed (Fig 1C, 1D and 1E) [35], the time series from multi-electrode recording was divided into 5 consecutive 10-second time windows [15]. The duration of the observation windows (10 seconds) is sufficiently larger than the cycle length of human AF (120–240 msec; 250–500 bpm) [33] by two orders of magnitude. With this time scale, there is typically no consistent pattern of activation during human AF. We manually inspected individual electrograms to confirm this claim. Because there was no consistent pattern of activation during the observation window, our method focused on probability distributions of activation instead of the specific direction or geometry of the activation wavefront.

Mutual information *I* (*X; Y*) was calculated from time series *X* and *Y* between each pair of electrodes within the 64-lead basket catheter to quantify pairwise information sharing in each time window [15,36].

$$\begin{array}{l} I(X;Y)=\sum_{x,y}p(x,y)\;\log\frac{p(x,y)}{p(x)p(y)}\\ \;=H(X)+H(Y)-H(X,Y) \end{array}$$ (1)

In this paper we will always use natural logarithms with a base of *e* and the resulting information measures will have units of the natural unit of information (nat).

**Fig 2:**
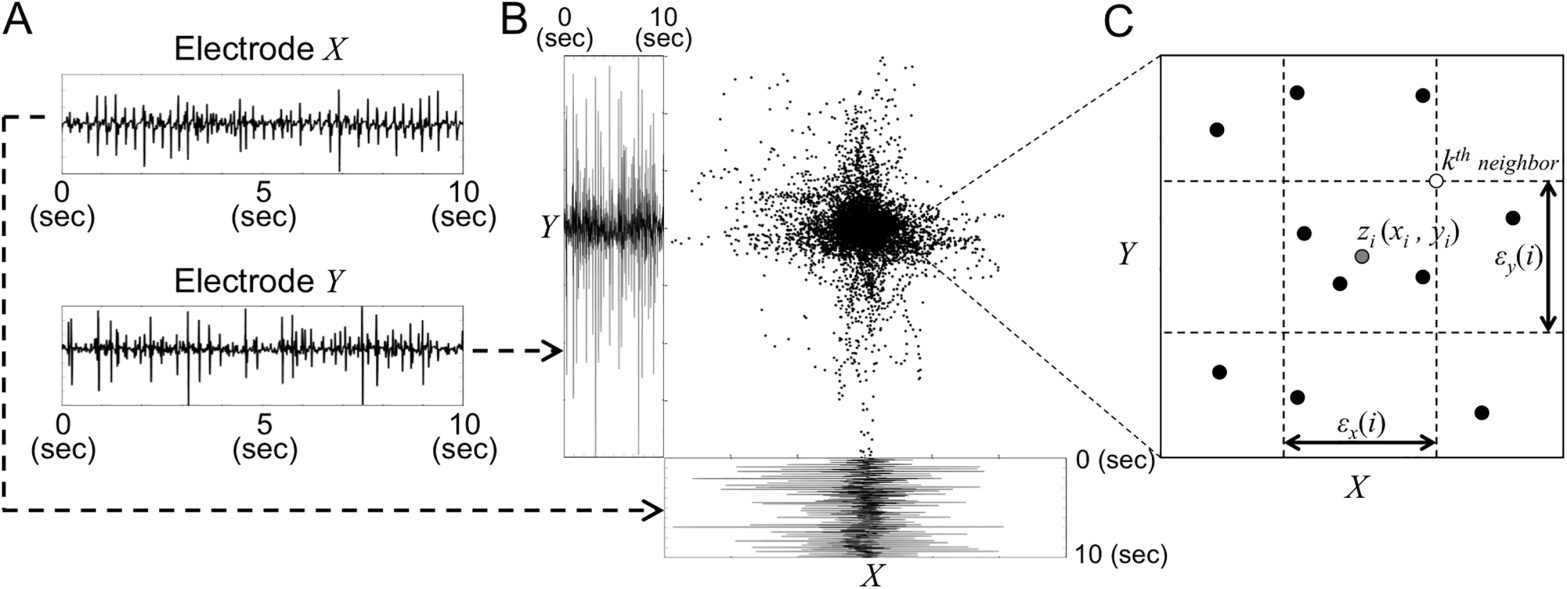
Mutual information estimation by *k*-nearest neighbor statistics in the joint space. ***A*.** *Preprocessed time series within the observation window (10 seconds) at electrode X and Y;* ***B*.** *Two-dimensional (2-D) joint space Z =* (*X*, *Y*)*;* ***C***. *Determination of ε*_*x*_(*i*), *ε*_*y*_(*i*), *n*_*x*_(*i*) and *n*_*y*_(*i*) *for a sample z*_*i*_ *(x*_*i*_, *y*_*i*_*)*. A case for *k = 4* is shown.

In Eq 1, *p*(*x*) and *p*(*y*) denote the probability density function of the time series generated by *X* and *Y*, respectively, and *p*(*x*, *y*) denotes the joint probability density function of *X* and *Y*. *H* (*X*) and *H* (*Y*) denote the Shannon entropy of *X* and *Y*, and *H* (*X*, *Y*) denotes the joint entropy of *X* and *Y*. To estimate Eq 1 from the electrode data, we use the *k*-nearest neighbor statistics algorithm proposed by Kraskov *et al*. [36]. The algorithm improves on traditional box kernel estimators by integrating the Kozachenko-Leonenko estimators of log-probabilities, bias correction, and variable kernel bandwidth to assist with the density of samples, which in turn smooth out errors in the probability density function estimation. The algorithm proceeds as follows. First, given a pair of preprocessed time series *X* and *Y* (Fig 2A), we construct a two-dimensional (2-D) joint space *Z =* (*X*, *Y*) (Fig 2B). For each sample *z*_*i*_ *=* (*x*_*i*_, *y*_*i*_) in this joint space, we find its *k*^th^ nearest neighbor using max norms to compute distance in the *X* and *Y* directions. In this paper we used *k = 4* according to the analysis by Kraskov *et al*. [36]. We then define *ε*_*x*_(*i*) and *ε*_*y*_(*i*) as the projection of distance between the sample and its *k*^th^ nearest neighbor into the *X* and *Y* subspaces, and count the numbers, *n*_*x*_(*i*) and *n*_*y*_(*i*), respectively, of neighbors within and on the boundaries of each marginal space (Fig 2C). In particular, *n*_*x*_(*i*) is the number of points *x*_*j*_, where ||*x*_*i*_−*x*_*j*_||≤ *ε*_*x*_(*i*)/2, and similarly the number *n*_*y*_(*i*) is the number of points *y*_*j*_, where ||*y*_*i*_−*y*_*j*_||≤ *ε*_*y*_(*i*)/2 (Fig 2C). Mutual information is then estimated by

$$I(X;Y)=\varphi (k)-\frac{1}{k}-\frac{1}{N}\sum_{i=1}^{N}\left[\varphi (n_{x}(i))+\varphi (n_{y}(i))\right]+\varphi (N)$$ (2)

where *ψ(x)* is the digamma function. Note that we only need to consider an integer domain of the digamma function.

$$\varphi (n)=-\gamma +\sum_{j=1}^{n-1}\frac{1}{k}$$ (3)

where *γ* is the Euler-Mascheroni constant. The algorithm above by Kraskov *et al*. [36] is implemented in an open-source library (Java Information Dynamics Toolkit; http://jlizier.github.io/jidt/) [37]. We used custom Matlab code to adapt the library to calculate mutual information in our data set.

**Fig 3:**
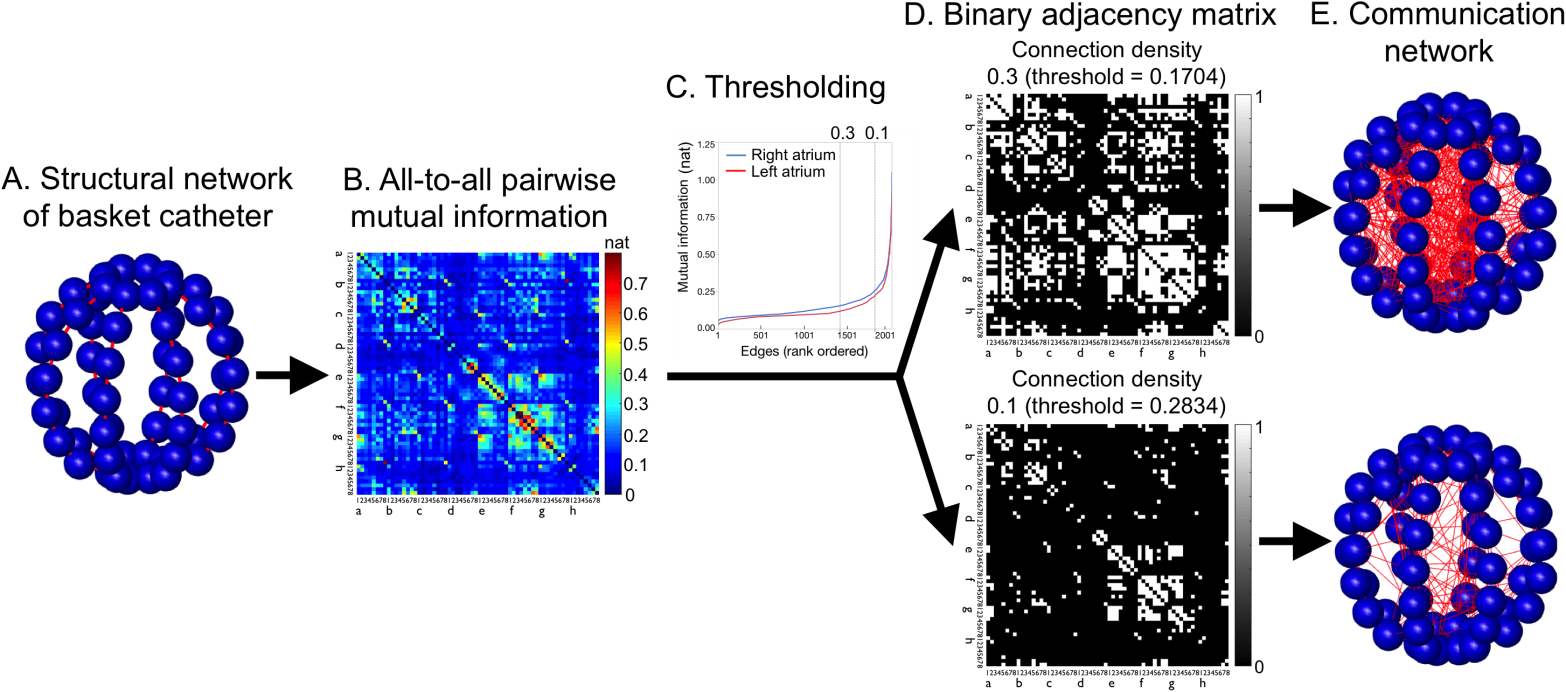
Communication network analysis. ***A***. *Structural network of basket catheter*. Edges (red line) indicate physical connectivity between the nodes (blue spheres; cardiac tissues adjacent to the electrodes). ***B***. *Representative average all-to-all mutual information matrix (64 x 64) of 5 consecutive 10-second time windows within the left atrium before ablation*. The *x*- and *y*-axes indicate individual electrodes of the basket catheter. The value of mutual information between two electrodes is color-coded and expressed in *nats*, the natural unit of information. The diagonal components from the upper left to the lower right are intentionally set to zero to exclude self-edges. ***C***. *Representative distribution of edges rank-ordered by mutual information*. In the absence of self-edges, there are 2,016 undirected edges between each pair of 64 electrodes (= [64 x 64–64]/2). To evaluate patient-specific parameters of communication, thresholds of mutual information are applied to set the connection density between 0.1 and 0.3, which preserves 10% to 30% of the strongest edges [30]. Blue, right atrium; red, left atrium. ***D***. *Binary adjacency matrix*. The top panel indicates a matrix with connection density 0.3 (threshold = 0.1704); the bottom panel indicates a matrix with connection density 0.1 (threshold = 0.2834). If the element (*i*, *j*) is one (white), an edge between electrode *i* and *j* is said to exist; otherwise (black), it does not exist. ***E***. *Communication network*. Edges (red line) indicate functional connectivity with suprathreshold mutual information between the nodes (blue spheres; cardiac tissues adjacent to the electrodes). The top panel indicates a communication network with connection density 0.3; the bottom panel indicates a communication network with connection density 0.1.

The average all-to-all mutual information matrix (64 x 64) of the 5 consecutive 10-second time windows was converted to binary adjacency matrices for the communication networks consisting of the nodes (= cardiac tissues adjacent to the electrodes) and undirected edges (= shared information) between nodes by applying a range of thresholds (Fig 3) [38–40]. For example, if mutual information between two electrodes is equal to or exceeds a threshold, an edge is said to exist; otherwise, it does not exist. Self-edges were excluded. To evaluate patient-specific parameters of communication, thresholds of mutual information were applied to set the connection density between 0.1 and 0.3, which preserved 10% to 30% of the strongest edges [30]. This enabled comparison of the communication network topology irrespective of the overall between-group difference in the weights and across a range of network connectivity [38].

### Communication network analysis

To assess the functional connectivity of communication networks during AF, we evaluated six network parameters: number of edges, average degree, giant component, average clustering coefficient, characteristic path length, and small-world index. The *number of edges* is the total number of edges present in a network. The *average degree* is the average number of edges connected to a node in a network, which is obtained by dividing the number of edges by the total number of nodes in a network. The *giant component* of a network is the largest connected subgraph in which all pairs of nodes are connected by at least one path. The number of nodes in the giant component therefore provides a simple measure of how well connected a network is. The *average clustering coefficient* of a network is the average of the clustering coefficients [25] of all the nodes and is equal to the probability that two neighbors of a given node are neighbors themselves, which quantifies local connectivity. A large clustering coefficient is associated with a high level of redundancy in the paths through the network and thus often serves as a measure of network resiliency. The average clustering coefficient is normalized by random networks [41] with the same number of degrees and edges. The *characteristic path length* of a network is the average of the geodesic path lengths (the number of edges contained in the shortest path connecting two nodes) between all pairs of nodes, which quantifies global connectivity. The characteristic path length is also normalized by random networks. The *small-world index* is the ratio of the normalized average clustering coefficient over the normalized characteristic path length, which quantifies network efficiency and robustness [42]. The small-world index was expressed in normal z standard deviation units (z-score).

### Statistical analysis

Continuous variables are expressed as mean ± standard deviation (SD) if normally distributed or otherwise as median (interquartile range [IQR]; 25th-75th percentile); categorical variables are expressed as frequencies and percentages. A two-sided p-value of <0.05 was considered significant. To compare network parameters, we used a Wilcoxon signed-rank test between groups. We used JMP Pro Version 12.1.0 (SAS Institute, Inc., Cary, NC) to perform all statistical analyses.

## Results

### Clinical characteristics

**Fig 4:**
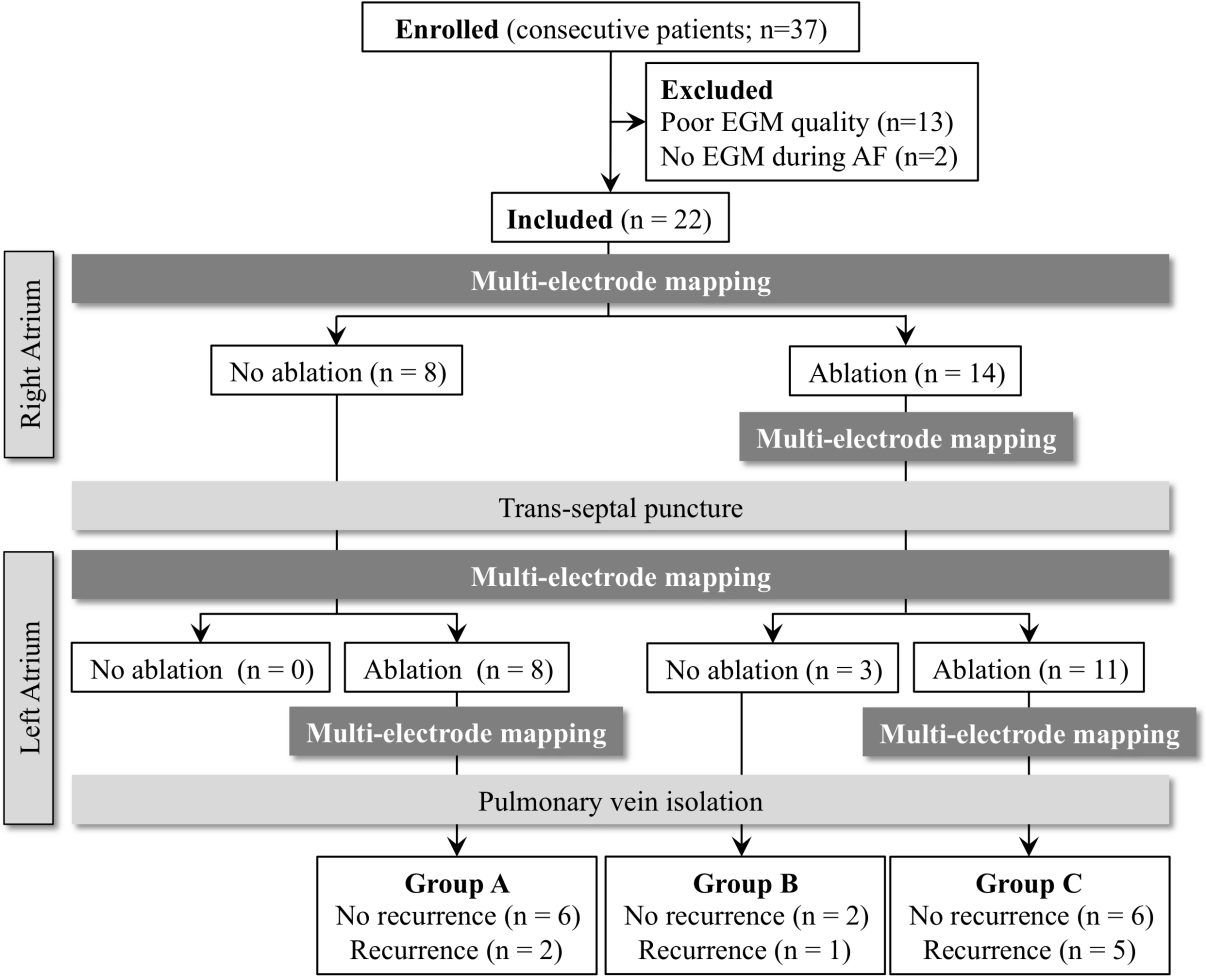
Patient enrollment. EGM, electrogram.

**Fig 5:**
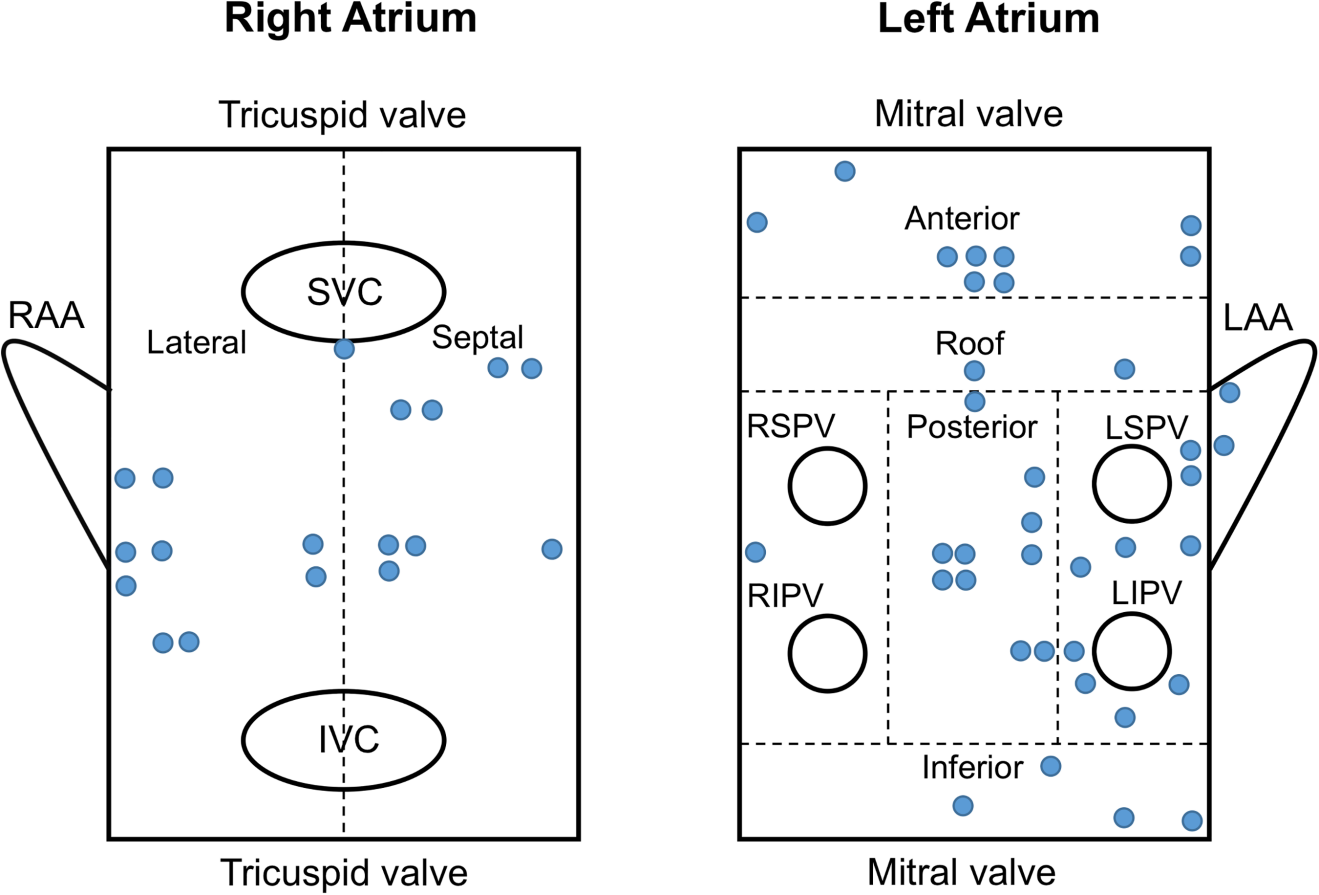
Ablation sites guided by the focal impulse and rotor modulation mapping system. Blue circles indicate ablation sites. SVC, superior vena cava; IVC, inferior vena cava; RAA, right atrial appendage; LAA, left atrial appendage; RSPV, right superior pulmonary vein; LSPV, left superior pulmonary vein; RIPV, right inferior pulmonary vein; LIPV, left inferior pulmonary vein.

All the patients presented to the electrophysiology laboratory in AF. Thirteen patients were excluded because of poor signal quality of multi-electrode electrograms. Two patients were excluded because they were in sinus rhythm or atrial flutter during multi-electrode mapping post-procedure. The remaining 22 patients (63.5 ± 9.7 years, 78% male) were included in the final analysis (Fig 4). Patient demographics are summarized in Table 1. The duration of time since AF diagnosis was 4.4 ± 3.7 years, and all patients had persistent AF. Ten patients (45%) had undergone previous AF ablation procedures. The patients were divided into three subgroups (Fig 4). In Group A (n = 8), the patients underwent FIRM-guided ablation only in the left atrium; in Group B (n = 3), the patients underwent FIRM-guided ablation only in the right atrium; in Group C (n = 11), the patients underwent FIRM-guided ablation in both atria. FIRM-guided ablation sites are shown in Fig 5. After FIRM-guided ablation and multi-electrode mapping, the entrance and exit block of all pulmonary veins were achieved in all patients. In 3 patients, acute termination of AF was observed. In one patient in Group A, FIRM-guided ablation converted AF into sinus rhythm. In another patient in Group A, FIRM-guided ablation converted AF into atypical atrial flutter. In one patient in Group C, FIRM-guided ablation converted AF into sinus rhythm. During the 6-month follow-up after the procedure, arrhythmic recurrence occurred in 8 patients (36%): Group A (n = 2), Group B (n = 1), and Group C (n = 5). There was no difference in clinical characteristics between patients with and without recurrence, including age, left atrial (LA) diameter, chambers of ablation, and total ablation time (Table 1).

**Table 1:** Patient demographics. Data are presented as mean ± standard deviation or n (%). P-value was calculated between patients with recurrence and no recurrence using Pearson's *χ*^2^ test for categorical variables and Student’s *t*-tests for continuous variables. AF, atrial fibrillation; CHA_2_DS_2_-VASc, combined stroke risk score: Cardiac failure, Hypertension, Age ≥65 or 75 years, Diabetes, prior Stroke/ transient ischemic attack (TIA), VAscular disease, Sex category; LA, left atrial; LV, left ventricular.

	Total (n = 22)	Recurrence (n = 8)	No recurrence (n = 14)	P Value
Age, years	63.5 ± 9.7	58.5 ± 9.0	66.3 ± 9.2	0.06
Sex, male	17 (78)	7 (88)	10 (71)	0.37
Body mass index, kg/m^2^	29.5 ± 5.7	30.4 ± 3.4	29.0 ± 6.8	0.56
Duration of AF, years	4.4 ± 3.7	4.9 ± 5.3	4.0 ± 2.5	0.57
Redo procedures	10 (45)	3 (38)	7 (50)	0.57
CHA_2_DS_2_-VASc score	1.9 ± 1.1	1.4 ± 1.2	2.2± 1.0	0.30
Underlying disease				
Heart failure	3 (14)	1 (13)	2 (14)	0.91
Hypertension	14 (64)	4 (50)	10 (71)	0.14
Diabetes	2 (9)	0 (0)	2 (14)	0.11
TIA / stroke	1 (5)	0 (0)	1 (7)	0.17
Echocardiography				
LV ejection fraction, %	52.5 ± 8.6	51.3 ± 10.6	53.3 ± 7.3	0.72
LA diameter, mm	46.9 ± 8.7	48.3 ± 1.0	46.0 ± 0.8	0.66
Ablation procedure				
RFCA in right atrium	14 (64)	6 (75)	8 (57)	0.79
RFCA in left atrium	19 (86)	7 (88)	12 (86)	0.36
Total RFCA time, min	45.5 ± 18.3	42.5 ± 25.0	47.0 ± 14.8	0.59

### Communication networks between right and left atrium

**Fig 6:**
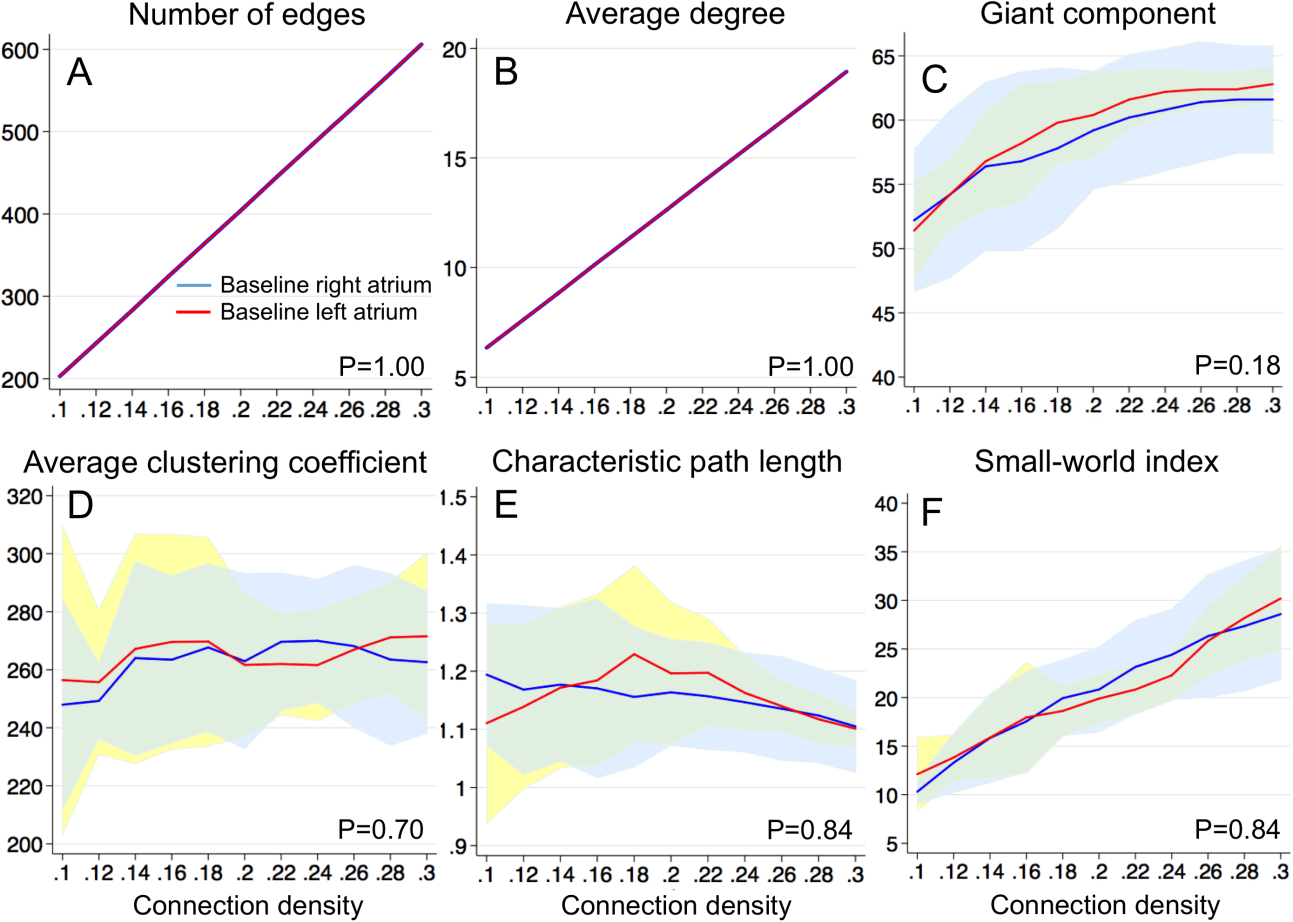
Baseline communication network parameters prior to ablation between the right and the left atrium. ***A***. *Number of edges*, ***B***. *Average degree*, ***C***. *Nodes in giant component*, ***D***. *Average clustering coefficient*, ***E***. *Characteristic path length*, ***F***. *Small-world index*. Baseline right atrium–solid blue line (mean) + 95% confidence interval (CI) (light blue); baseline left atrium–solid red line (mean) + 95%CI (light yellow).

Group A (n = 8) provided samples to compare communication networks during AF between the atria prior to ablation within the same patients. There was no significant difference between the atria in all the communication network parameters studied (Fig 6). Since the communication network parameters were normalized to the connection density, the number of edges (Fig 6A, P = 1.00) was identical between both atria. The average degree (Fig 6B, P = 1.00) was also identical between both atria because the number of nodes remained constant (= 64 in this study). There was no significant difference between the atria in the number of nodes in the giant component (Fig 6C, P = 0.18), the average clustering coefficient (Fig 6D, P = 0.70), the characteristic path length (Fig 6E, P = 0.84), and the small-world index (Fig 6F, P = 0.84). These results indicate that the functional connectivity of communication networks during AF was equivalent in both atria.

### Impact of ablation on communication networks

**Fig 7:**
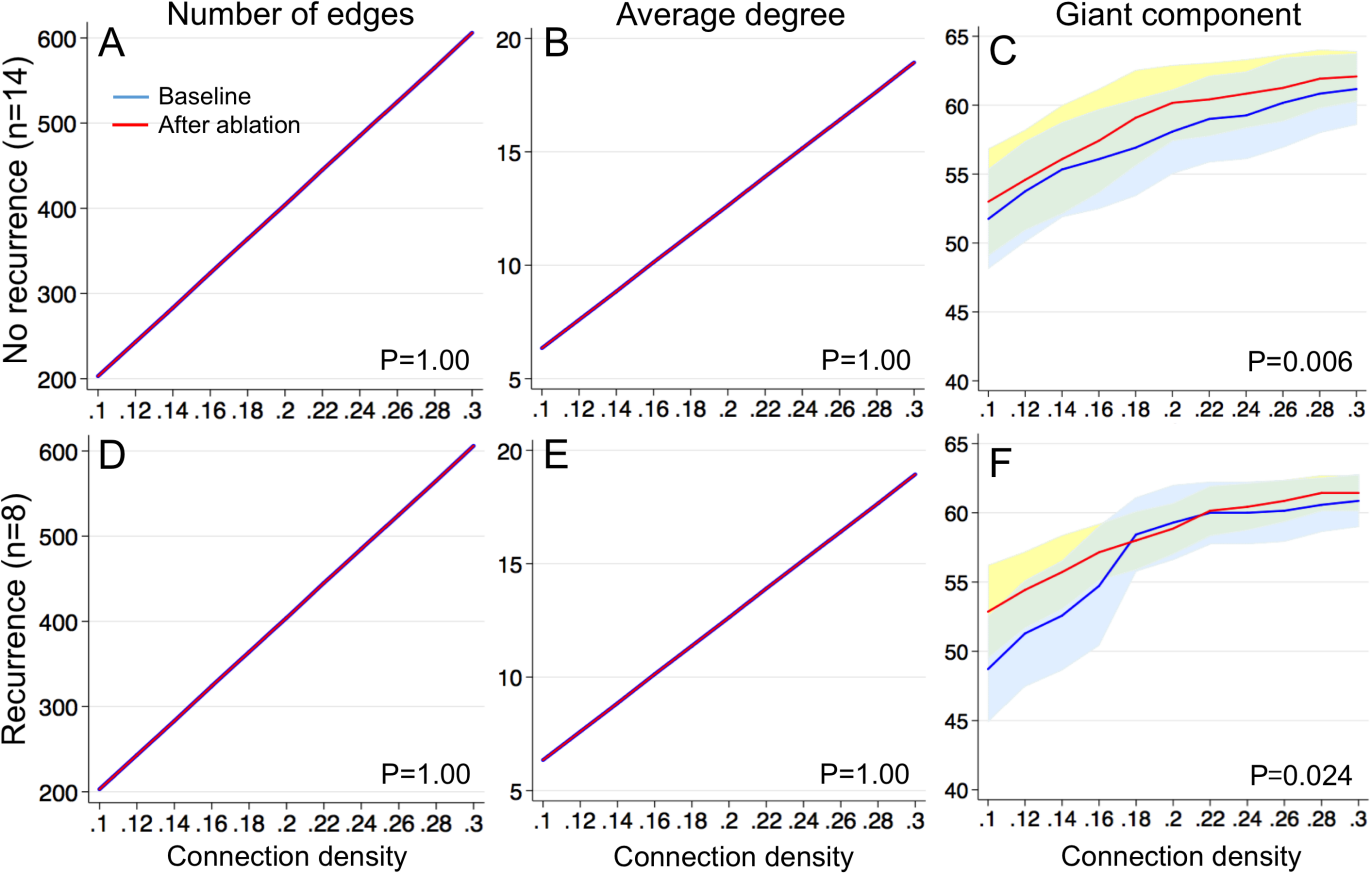
Impact of ablation on basic communication network parameters. *Number of edges* (A, D), *Average degree* (B, E), and *Giant component* (C, F). The baseline right atrium–solid blue line (mean) + 95% confidence interval (CI) (light blue); left atrium after ablation–solid red line (mean) + 95%CI (light yellow).

Because there was no significant difference in the functional connectivity of communication networks during AF in both atria, we compared the communication network parameters between the baseline multi-electrode mapping of the right atrium and the final multi-electrode mapping of the left atrium prior to PVI to evaluate the impact of ablation on the functional connectivity of communication networks (Fig 7). This allowed comparison of communication networks during AF between the baseline functional connectivity of communication networks and after ablation in all the patients studied (n = 22).

**Fig 8:**
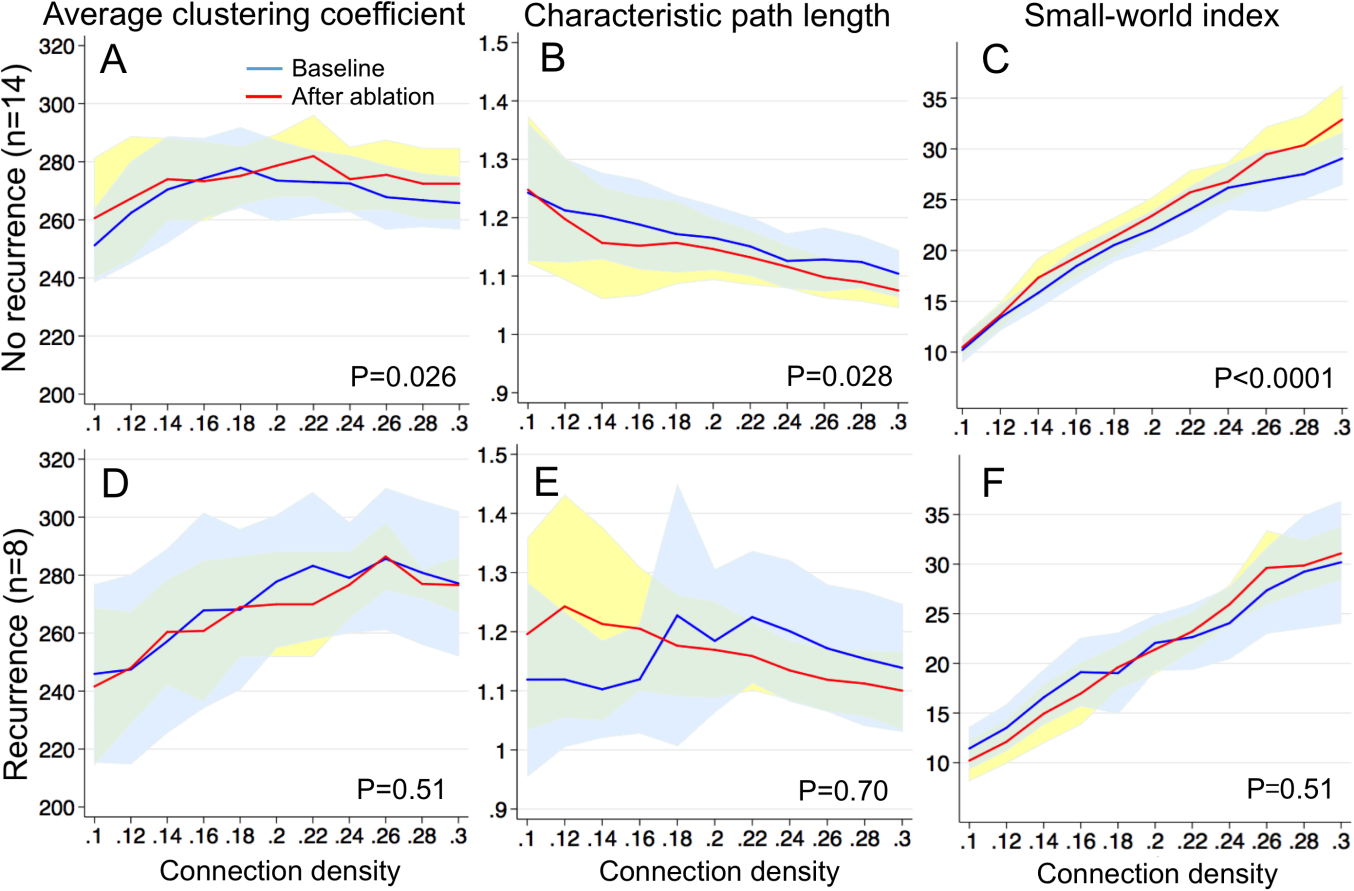
Impact of ablation on local and global connectivity of communication networks. *Average clustering coefficient* (A, D), *Characteristic path length* (B, E), and *Small-world index* (C, F). The baseline right atrium–solid blue line (mean) + 95% confidence interval (CI) (light blue); left atrium after ablation–solid red line (mean) + 95%CI (light yellow).

As mentioned above, since the communication network parameters were normalized to the connection density, the number of edges (Fig 7A and 7D, P = 1.00 each) and the average degree (Fig 7B and 7E, P = 1.00 each) were identical between the baseline and after ablation. The number of nodes in the giant component was significantly higher after ablation than the baseline in patients without (Fig 7C, P = 0.006) and with recurrence (Fig 7F, P = 0.024). This indicates that ablation effectively rewired the communication networks to improve the overall connectivity with the same number of edges and nodes. The average clustering coefficient significantly increased after the ablation in patients with no recurrence (Fig 8A; P = 0.026). The average clustering coefficient did not significantly change in patients with recurrence (Fig 8D; P = 0.51).

These results indicate that ablation improved the local connectivity of communication networks in patients with no recurrence. The characteristic path length significantly decreased after ablation in the patients with no recurrence (Fig 8B; P = 0.028). The characteristic path length did not significantly change in patients with recurrence (Fig 8E; P = 0.70). These results indicate that ablation improved the global connectivity of communication networks in patients with no recurrence. The small-world index significantly increased after ablation in the patients with no recurrence (Fig 8C; P<0.0001). The small-world index did not significantly change in the patients with recurrence (Fig 8F; P = 0.51). These results indicate that ablation improved the communication network efficiency and robustness in the patients with no recurrence. Furthermore, Group A (n = 8) also provided samples to compare communication networks during AF within the same atrium (left atrium) at baseline and after ablation, where the same impact of ablation on communication network parameters was observed (Figure A in S1 File, Supporting information).

### Communication network connectivity and acute termination of atrial fibrillation during ablation

**Fig 9:**
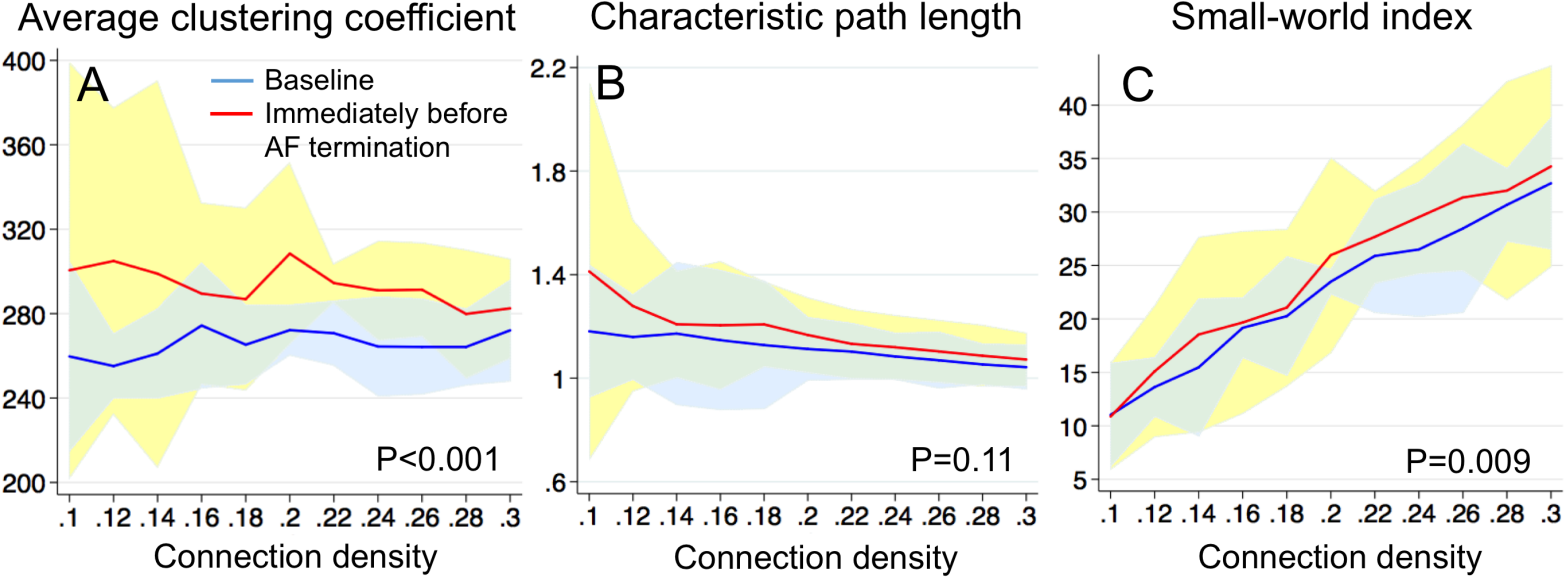
Communication network connectivity and acute termination of atrial fibrillation. ***A***. *Average clustering coefficient*, ***B***. *Characteristic path length*, ***C***. *Small-world index*. Baseline right atrium–solid blue line (mean) + 95% confidence interval (CI) (light blue); left atrium immediately before atrial fibrillation (AF) termination–solid red line (mean) + 95%CI (light yellow).

To evaluate the change in the functional connectivity of the communication networks that are associated with acute termination of AF during ablation, we compared the communication network parameters between the baseline multi-electrode mapping of the right atrium and the multi-electrode mapping immediately prior to acute termination of AF (n = 3, Fig 9). The average clustering coefficient significantly increased compared with the baseline (Fig 9A, P<0.001). In contrast, the characteristic path length did not significantly change between the baseline and immediately before AF termination (Fig 9B, P = 0.11). The small-world index significantly increased compared with the baseline (Fig 9C, P = 0.009). These results suggest that improvement in small-world attributes of the communication networks by ablation can lead to synchronization.

## Discussion

In this study, we defined the communication networks during persistent AF using information sharing to evaluate functional connectivity. Our aim was to quantify the similarity of activation patterns between the electrodes over a sufficiently long observation window relative to the cycle length of human AF. We chose to use mutual information because the joint probability distribution of activation patterns between the electrodes is inherently incorporated in it. An advantage of our analysis lies in its simplicity. It allows reconstruction of communication networks by focusing on the probability distributions of activation patterns without a need to determine the specific direction or geometry of activation wavefronts, which is challenging in human AF. Information theory metrics such as Shannon entropy [35,43] and Kolmogorov entropy [44] have been used in the past to quantify cardiac fibrillation in humans, but to our knowledge, this is the first study to use mutual information to quantify the communication between cardiac tissues in human AF. Zahid *et al*. [45] used a network approach to analyze human left atrial flutter, but their network construction was based on numerical simulation with a Courtemanche human atrial cell model [46] instead of human data. In addition, they used a network approach to estimate the optimal target of ablation, rather than to quantify communication within the atria. Furthermore, the arrhythmia of interest was left atrial flutter with a fixed anatomical reentry circuit, whereas we are interested in AF with rapidly changing activation patterns. A network approach was also used in other studies to analyze cardiac fibrillation [47–49]; however, in those studies the network construction was also based on numerical simulation using a Fenton-Karma model of cardiac tissue [50] and the Courtemanche model [46]. In addition, they used a network approach to assess the association between percolation thresholds and microreentry that triggers fibrillation in the presence of fibrosis, rather than to quantify the communication within the atria. Therefore, to our knowledge, this is the first study to utilize a network approach to quantitatively analyze the functional communication networks of human AF.

### Main findings

We find that there is no significant difference in any of the communication network parameters studied between both atria at baseline. This important finding suggests that persistent AF—despite its seemingly diverse and chaotic electrocardiographic phenotypes—may have a fairly uniform communication network topology that is amenable to quantitative analysis. We also find that ablation effectively rewires the communication network of persistent AF. Ablation improves the overall connectivity with the same number of edges and nodes (Fig 7C and 7F, Giant component). In addition, successful ablation improves the local connectivity by increasing the average clustering coefficient (Fig 8A). Successful ablation also improves the global connectivity by decreasing the characteristic path length (Fig 8B). As a result, successful ablation improves the efficiency and robustness of the communication network by increasing the small-world index (Fig 8C). These changes are not observed in patients with AF recurrence. Furthermore, a significant increase in the small-world index after ablation is associated with synchronization of the rhythm by acute AF termination.

## New insights into atrial fibrillation

In this study, we provide evidence that ablation effectively rewires the functional connectivity of the communication networks of persistent AF, making the network more robust and efficient. Our results have two important clinical implications for AF. First, our analysis sheds new light on the mechanism of AF. We find that patients with persistent AF have similar communication network characteristics in both atria, and that localized ablation in the right and/or left atrium improves not only the local connectivity but also the global connectivity. This finding supports the localized source hypothesis that AF is sustained by spatially localized sources such as organized reentrant circuits (“*rotors*”) [51,52] or focal impulses [53], rather than by homogeneously distributed multiple electrical waves [54]. Second, our analysis provides a new approach to quantifying AF. We quantify the communication network of AF, rather than simply the presence or absence of AF. Previous studies [4,55] have relied on a binary definition of AF; individuals either have or do not have AF. The binary definition of AF may be both insensitive, missing an active but non-inducible tendency to sustain AF [56], and simplistic, failing to capture important features that contribute to the maintenance of AF. Our findings indicate that quantitative analysis of the communication networks of AF provides patient-specific diagnostic parameters that could potentially serve as a valid endpoint for therapeutic interventions. In addition, because quantitative analysis of the communication networks can be performed in individuals without clinically recognized AF, it could potentially help identify individuals at high risk of developing AF, such as those with heart failure.

### Implications for other complex networks

Identification of general principles linking the structure and function in complex networks has been a recurring theme. Our results demonstrate that AF is associated with a reduction in the small-world attributes of the communication networks. This finding indicates a possibility that AF and brain diseases associated with disruptions of small-world organization, such as Alzheimer's disease [57,58] and schizophrenia [29,30] may share the common pathologic patterns of functional interactions. Furthermore, improvement in the small-world index led to synchronization of the rhythm by acute termination. Synchronization can have both physiological [59–62] and pathological [63,64] impact on brain functions, but insights on the structure-function analysis obtained from the cardiac networks may contribute to understanding the mechanism of cognitive and behavioral diseases and to the development of therapeutic strategies. For many years, neuroscientists have taken advantage of network analysis to address the integrative nature of brain function [40]; however, application of network analysis to neuroscience is almost exclusively limited to theoretical considerations. Similar to the brain, the heart is a complex network of dynamical systems, where the topology of structural networks can be intentionally altered by standard therapeutic interventions. Our study demonstrates the strength of the heart as an empiric platform to evaluate targeted structural perturbations as a method of network optimization for improved understanding of the structure-function relationship of complex networks.

### Limitations

There are several limitations to this study. First, this was a singe-center, retrospective study with a relatively small sample size. In addition, we excluded 35% of enrolled patients (13 out of 37) due to poor electrogram quality. Therefore, there is a non-negligible chance of selection bias. Our results should be confirmed in a larger prospective study. Second, the recurrence was defined at 6 months, which is shorter than the standard definition at 12 months [3]. However, all patients in the study had persistent AF, and ten patients (45%) who had undergone previous AF ablation had developed recurrence within 6 months. Therefore, we believe the current definition of recurrence is valid in our context. Third, our analysis might have been affected by the location of the basket catheter within the heart chamber [35]. However, our results showed consistent trends of each network parameter at baseline or after ablation in both atria even though the measurements in different heart chambers were acquired at different time points. Therefore, communication network analysis with local and global connectivity appears to be spatially and temporally robust. Fourth, recurrence could have been affected by PVI as well as by FIRM-guided ablation. However, all patients with or without recurrence underwent PVI with demonstrated entrance and exit block of all pulmonary veins. In addition, all patients had persistent AF, for which PVI alone is associated with high recurrence rates. Therefore, the difference in the outcome in this study is likely derived from FIRM-guided ablation. Finally, we examined the parameters of undirected, thresholded networks. More complex communication network analysis may be possible using directed and weighted networks using directed measures of communications such as transfer entropy [65]. However, because there was no consistent directionality of activation that persists over the entire observation window, rather than focusing on the direction of information flow using a directed measure of connectivity, we focused on assessment of the joint probability distribution of activation using mutual information.

### Conclusions

Successful ablation rewires the communication network during persistent AF, making it more robust, efficient, and easier to synchronize. Quantitative analysis of the communication networks of AF provides not only a mechanistic insight that AF may be sustained by spatially localized sources and global functional connectivity, but also patient-specific diagnostic parameters that could potentially serve as a valid endpoint for therapeutic interventions. In addition, the structure-function analysis obtained from the heart can contribute to understanding the mechanism of cognitive and behavioral diseases and to the development of therapeutic strategies in the brain networks. Furthermore, our analysis provides evidence linking targeted structural perturbations and functional improvement in complex networks. Our understanding of the structure-function relationship of complex networks can be improved by evaluating targeted structural perturbations in the heart as a method of network optimization.

## Supporting information

S1 File The supporting information including text, one figure, and references. (DOCX)
