# Supplementary material for: Ablation as targeted perturbation to rewire communication network of persistent atrial fibrillation
Source: PLoS One. 2017 Jul 5;12(7):e0179459. doi: 10.1371/journal.pone.0179459 (PMC5497967; doi:10.1371/journal.pone.0179459)
Supplement: S1 File — (DOCX) [file pone.0179459.s001.docx]

Supporting Information for

**Ablation as Targeted Perturbation to Rewire**

**Communication Network of Persistent Atrial Fibrillation**

Susumu Tao, Samuel F. Way, Joshua Garland, Jonathan Chrispin, Luisa A. Ciuffo,

Muhammad A. Balouch, Saman Nazarian, David D. Spragg, Joseph E. Marine,

Ronald D. Berger, Hugh Calkins, Hiroshi Ashikaga*

*To whom correspondence should be addressed

E-mail: [hashika1@jhmi.edu](mailto:hashika1@jhmi.edu)

**Appendix. Detailed Methods**

*Patient population.* We enrolled 37 consecutive patients in the study who underwent catheter ablation of persistent atrial fibrillation (AF) with multi-electrode mapping at the Johns Hopkins Hospital between January 2015 and April 2016. Persistent AF was defined as sustained AF that fails to self-terminate or requires electrical or chemical cardioversion after > 7 days [1]. The protocol was approved by the Johns Hopkins Medicine Institutional Review Board and all participants provided written informed consent.

*Multi-electrode mapping and catheter ablation.* All patients underwent pre-procedural transesophageal echocardiogram to rule out intracardiac thrombus. A 5-Fr quadripolar catheter was introduced from the femoral vein into the superior vena cava as the reference for unipolar recordings from multi-electrode mapping. The right atrial (RA) shell was created by a three-dimensional (3-D) electroanatomical mapping system (CARTO3; Biosense Webster, Inc., Diamond Bar, CA) with a 3.5-mm-tip, open-irrigated, force-sensing ablation catheter (ThermoCool SmartTouch; Biosense Webster, Inc.). A 64-electrode basket catheter (50 mm or 60 mm; Abbott Electrophysiology, Menlo Park, CA; Fig 1A) was advanced from the femoral vein through an 8.5F SL1 sheath (Daig Medical, Minnetonka, MN) to the right atrium. The basket catheter was manipulated to optimize electrode contact with the RA surface and maximize coverage of the entire chamber (Fig 1B). In patients presenting in sinus rhythm, AF was induced via rapid RA burst pacing. Induced AF was mapped after > 15 min [2]. Unipolar electrograms from the basket catheter were filtered at 0.05 Hz to 500 Hz and recorded during AF for 60 seconds at the sampling frequency of 977 Hz (Cardiolab; GE Healthcare, Waukesha, WI). The unipolar electrograms were analyzed using the RhythmView workstation (Abbott Electrophysiology), and patients underwent radiofrequency (RF) catheter ablation based on the focal impulse and rotor modulation (FIRM)-guided approach. The physiological rationale, algorithms, and approach of FIRM mapping have been described previously [2,3]. Ablation was performed using the force-sensing ablation catheter, targeting each area identified as a rotor center with power at the discretion of the operator (generally 25 W on the posterior wall and 30 W in the other regions). Ablation was continued until abatement of local electrograms, typically 15-30 seconds at each tip location. A repeat rotor map was obtained and any additional identified rotors were ablated [4]. The basket catheter was then advanced trans-septally to the left atrium, and multi-electrode mapping and ablation were repeated. After completion of the FIRM-guided ablation, pulmonary vein isolation was performed using wide area circumferential ablation of the pulmonary vein antra until the entrance and exit block was demonstrated for each pulmonary vein. Intravenous heparin was infused to achieve an activated clotting time > 350 seconds in all cases to minimize the risk of intracardiac thrombosis during the procedure.

*Clinical follow-up*. Arrhythmic recurrence was defined as AF, atrial tachycardia, or atrial flutter of at least 30-second duration after a three-month blanking period [5]. At each in-office visit scheduled at 3 months after the ablation procedure, a physical examination and a 12-lead electrocardiogram (ECG) were performed. If symptoms suggestive of an arrhythmia occurred, patients were asked to undergo a 24-hour Holter monitor or a 30-day event monitor. Recurrence was defined at 6 months post-procedure.

*Preprocessing*. We performed data analysis using MATLAB R2016a (MathWorks, Inc., Natick, MA). We used a preprocessing method similar to the one proposed by Benharash *et al.* (Fig 1C, D, E) [6]. The raw unipolar electrograms from the multi-electrode mapping were filtered at 15 to 100 Hz to remove the baseline drift and noise. The mean QRS-T complex was computed from all the QRS complexes during the entire recording period. The mean QRS–T complex was subtracted from each segment to remove the far-field ventricular electrogram, leaving only the atrial electrogram for each of the 64 channels.

*Mutual information*. The time series from multi-electrode recording was divided into 5 consecutive 10-second time windows. Mutual information *I* (*X; Y*) was calculated from the time series processes *X* and *Y* between each pair of electrodes to quantify the pairwise information sharing in each window [7].

$I\left( X;Y \right)= \sum_{x,y} p(x,y)\log\frac{p(x,y)}{p\left( x \right)p(y)}$ (1)

where *p*(*x*) and *p*(*y*) denote the probability density function of the time series generated by *X* and *Y* , respectively, and *p*(*x, y*) denotes the joint probability density function of *X* and *Y* . We used the mutual information estimator proposed by Kraskov *et al*. [8] implemented in the Java Information Dynamics Toolkit (JIDT) [9] to calculate the mutual information between each pair of electrodes (Fig 2A). The unit of mutual information is the natural unit of information (*nat*) based on natural logarithms and powers of *e*. The details of mutual information calculation are described in the *Materials and methods* section of the main text. An all-to-all mutual information matrix (64 x 64) was created from each of the 5 consecutive 10-second time windows, and the average mutual information matrix (Fig 2B) was obtained by element-wise averaging of the 5 matrices to minimize the impact of noise.

*Adjacency matrix of communication networks*. The average mutual information matrix was converted to a binary adjacency matrix ***A*** for the communication network consisting of nodes (= cardiac tissues adjacent to the electrodes) and undirected edges (= shared information) between nodes by applying a range of thresholds (Fig 2C, D, E) [10-12]. For example, if the mutual information between electrodes *i* and *j* is equal to or exceeds a threshold, an edge between *i* and *j* is said to exist; otherwise, it does not exist. The diagonal components of ***A*** from the upper left to the lower right is intentionally set to zero to exclude self-edges. To evaluate patient-specific parameters of communication, thresholds of mutual information were applied to set the connection density between 0.1 and 0.3, which preserved 10% to 30% of the strongest edges [13]. This enabled comparison of the structural pattern of networks irrespective of the overall between-group difference in the weights and across a range of network connectivity [10].

*Communication network analysis*. The *degree* *k_i_* of node *i* in a network is the number of edges connected to it.

$k_{i}=\sum_{j=1}^{N} A_{j}$ (2)

where *A_ij_* is component (*i*, *j*) of an adjacency matrix ***A***, and *N* is the number of nodes in the network (*N* = 64 in this study). The *number of edges* *m* in a network is

$m=\frac{1}{2}\sum_{i=1}^{N} k_{i}=\frac{1}{2}\sum_{i,j}^{N} A_{ij}$ (3)

The *average degree* is the average number of edges connected to a node in a network. The *giant component* of a network is the largest connected subgraph in which all pairs of nodes are connected by at least one path. The number of nodes in the giant component therefore provides a simple measure of how well connected a network is. The *clustering coefficient* is a standard network statistic that quantifies the degree to which a network is organized into densely connected clusters [14]. The clustering coefficient *c_i_* of node *i* is defined as the fraction of all possible edges that exist between the neighbors of *i*, the set of nodes *N*(*i*) adjacent to *i*.

$c_{i}=\frac{ʋ_{i}}{k_{i}(k_{i}-1)/2}$ (4)

where *ν_i_* is the number of edges between neighbors of *i*.

$ʋ_{i}=\frac{1}{2}\sum_{j,k\in N(i)} A_{jk}$ (5)

The *average clustering coefficient* of a network is the average of the clustering coefficients of all nodes and is equal to the probability that two neighbors of a given node are neighbors themselves, which quantifies the local connectivity. A large clustering coefficient is associated with a high level of redundancy in the paths through the network and thus often serves as a measure of network resiliency. The average clustering coefficient is normalized by the average of the same parameter in 1,000 random networks [15] with the same number of degrees and edges. The path length separating two nodes in a network is defined as the number of edges contained in the shortest path (*geodesic path*) connecting the two nodes. The *characteristic path length* of a network is the average of the geodesic path lengths between all pairs of nodes, which quantifies global connectivity. The characteristic path length is also normalized by the average of the same parameter in random networks. The *small-world index* is the ratio of the normalized average clustering coefficient over the normalized characteristic path length, which quantifies network efficiency and robustness [16]. The small-world index is expressed in normal z standard deviation units (z-score).

*Statistical analysis*. Continuous variables are expressed as mean ± standard deviation (SD) if normally distributed or otherwise as median (interquartile range [IQR]; 25th-75th percentile), and categorical variables are expressed as frequencies and percentages. A two-sided p-value of <0.05 was considered significant. To compare network parameters, we used Wilcoxon signed-rank test between groups. We used JMP Pro Version 12.1.0 (SAS Institute, Inc., Cary, NC) to perform all statistical analyses.

**Raw Data**

The Uniform Resource Locator of the raw data is as follows: https://osf.io/26bvm/.

**Figure A**

**Figure A. Impact of ablation on local and global connectivity of communication networks within the same chamber (left atrium).** *Average clustering coefficient* (a, d), *Characteristic path length* (b, e), and *Small-world index* (c, f). Baseline left atrium – solid blue line (mean) + 95% confidence interval (CI) (light blue); left atrium after ablation – solid red line (mean) + 95%CI (light yellow).

**References**

1. January CT, Wann LS, Alpert JS, Calkins H, Cigarroa JE, Cleveland JC, Jr., et al. 2014 AHA/ACC/HRS Guideline for the Management of Patients With Atrial Fibrillation: A Report of the American College of Cardiology/American Heart Association Task Force on Practice Guidelines and the Heart Rhythm Society. Circulation. 2014 Dec 2;130(23):e199-267. PubMed PMID: 24682347.

2. Narayan SM, Krummen DE, Shivkumar K, Clopton P, Rappel WJ, Miller JM. Treatment of atrial fibrillation by the ablation of localized sources: CONFIRM (Conventional Ablation for Atrial Fibrillation With or Without Focal Impulse and Rotor Modulation) trial. Journal of the American College of Cardiology. 2012 Aug 14;60(7):628-36. PubMed PMID: 22818076. Pubmed Central PMCID: 3416917.

3. Narayan SM, Krummen DE, Rappel WJ. Clinical mapping approach to diagnose electrical rotors and focal impulse sources for human atrial fibrillation. Journal of cardiovascular electrophysiology. 2012 May;23(5):447-54. PubMed PMID: 22537106. Pubmed Central PMCID: 3418865.

4. Chrispin J, Gucuk Ipek E, Zahid S, Prakosa A, Habibi M, Spragg D, et al. Lack of regional association between atrial late gadolinium enhancement on cardiac magnetic resonance and atrial fibrillation rotors. Heart rhythm. 2016 Mar;13(3):654-60. PubMed PMID: 26569460.

5. Calkins H, Kuck KH, Cappato R, Brugada J, Camm AJ, Chen SA, et al. 2012 HRS/EHRA/ECAS expert consensus statement on catheter and surgical ablation of atrial fibrillation: recommendations for patient selection, procedural techniques, patient management and follow-up, definitions, endpoints, and research trial design: a report of the Heart Rhythm Society (HRS) Task Force on Catheter and Surgical Ablation of Atrial Fibrillation. Developed in partnership with the European Heart Rhythm Association (EHRA), a registered branch of the European Society of Cardiology (ESC) and the European Cardiac Arrhythmia Society (ECAS); and in collaboration with the American College of Cardiology (ACC), American Heart Association (AHA), the Asia Pacific Heart Rhythm Society (APHRS), and the Society of Thoracic Surgeons (STS). Endorsed by the governing bodies of the American College of Cardiology Foundation, the American Heart Association, the European Cardiac Arrhythmia Society, the European Heart Rhythm Association, the Society of Thoracic Surgeons, the Asia Pacific Heart Rhythm Society, and the Heart Rhythm Society. Heart rhythm. 2012 Apr;9(4):632-96 e21. PubMed PMID: 22386883.

6. Benharash P, Buch E, Frank P, Share M, Tung R, Shivkumar K, et al. Quantitative analysis of localized sources identified by focal impulse and rotor modulation mapping in atrial fibrillation. Circulation Arrhythmia and electrophysiology. 2015 Jun;8(3):554-61. PubMed PMID: 25873718.

7. Ashikaga H, Aguilar-Rodriguez J, Gorsky S, Lusczek E, Marquitti FM, Thompson B, et al. Modelling the heart as a communication system. Journal of the Royal Society, Interface / the Royal Society. 2015 Apr 6;12(105). PubMed PMID: 25740854. Pubmed Central PMCID: 4387519.

8. Kraskov A, Stogbauer H, Grassberger P. Estimating mutual information. Physical review E, Statistical, nonlinear, and soft matter physics. 2004 Jun;69(6 Pt 2):066138. PubMed PMID: 15244698.

9. Lizier JT. JIDT: An information-theoretic toolkit for studying the dynamics of complex systems. Frontiers in Robotics and AI. 2014;1:11.

10. Bullmore ET, Bassett DS. Brain Graphs : Graphical Models of the Human Brain Connectome. Annu Rev Clin Psychol. 2011;7:113-40.

11. Rubinov M, Sporns O. Complex network measures of brain connectivity: uses and interpretations. NeuroImage. 2010 Sep;52(3):1059-69. PubMed PMID: 19819337.

12. Sporns O. Networks of the Brain. 1 ed: The MIT Press; 2010.

13. Rubinov M, Knock SA, Stam CJ, Micheloyannis S, Harris AW, Williams LM, et al. Small-world properties of nonlinear brain activity in schizophrenia. Human brain mapping. 2009 Feb;30(2):403-16. PubMed PMID: 18072237.

14. Watts DJ, Strogatz SH. Collective dynamics of 'small-world' networks. Nature. 1998 Jun 4;393(6684):440-2. PubMed PMID: 9623998.

15. Erdös P, Rényi A. On the evolution of random graphs. Publication of the Mathematical Institute of the Hungarian Academy of Sciences. 1960;5:17-61.

16. Humphries MD, Gurney K. Network 'small-world-ness': a quantitative method for determining canonical network equivalence. PloS one. 2008;3(4):e0002051. PubMed PMID: 18446219. Pubmed Central PMCID: 2323569.
